# Supplementary material for: The miR-100-5p Targets SMARCA5 to Regulate the Apoptosis and Intracellular Survival of BCG in Infected THP-1 Cells
Source: Cells. 2023 Feb 1;12(3):476. doi: 10.3390/cells12030476 (PMC9914254; doi:10.3390/cells12030476)
Supplement: Supplementary file 1 [file cells-12-00476-s001.zip › cells-2101062-supplementary.pdf]

Supporting Information

Table S1. qRT-PCR and PCR primer sequences.

Gene	Sequence 5′-3′	Products(bp)
Hsa-miR-100-5p	F: AACCCGTAGATCCGAACTTGTG	
Universal reverse	R: GCTGTCAACGATACGCTACGTAAC	
U6	F: CTCGCTTCGGCAGCACA<br>R: AACGCTTCACGAATTTGCGT	
SMARCA5	F: ATTACCGACACCGTAGAACAGA<br>R: GAGCCAGTTTAATCCTCGGAC	155
EPDR1	F：TGTGATATTGTCTACGCGGTTTT<br>R：GAGCAGTCTTCGCTCATCTTC	117
TRIB2	F: ATGAACATACACAGGTCTACCCC<br>R: GGGCTGAAACTCTGGCTGG	122
HS3ST3B1	F: GAAGACGCCCAGTTACTTCGT<br>R: CCTGTTTTTGAACGTCAAGCTCT	181
MTMR3	F: CTTTCTGACGTGGAGTTCGATT<br>R: GTTTGGGTAATACTCTGGGCATT	165
β-actin	F: CATGTACGTTGCTATCCAGGC<br>R: CTCCTTAATGTCACGCACGAT	250
SMARCA5 3'UTR	F: CCTCGAGGTCGAGGAAGAAAAAAGAAGCT<br>R: TTGCGGCCGCTGTTATTTGCAATTTTCTTTTTAAC	392
SMARCA5 3'UTR Mut	Fm: TCTTTAATTATGCCCACTTCATAAG<br>Rm: CTTATGAAGTGGGCATAATTAAAGA	392
HS3ST3B1 3'UTR	F: CCTCGAGCCGGGCTATGTACCTTACCC<br>R: TTGCGGCCGCAAAGGATGTGGATGTCCCTGTAA	580
HS3ST3B1 3'UTR Mut	Fm: TTGTTGTATGCCCATTTCAGCCT<br>Rm: AGGCTGAAATGGGCATACAACAA	580
EPDR1 3'UTR	F: CCTCGAGGCCTGTGCATAGGGAAGCG<br>R: TTGCGGCCGCAAATTCTTAAGTCTGGAAGGGATTT	564
EPDR1 3'UTR Mut	Fm: TGCATGCTGCCCATTACACAT<br>Rm: ATGTGTAATGGGCAGCATGCA	564
TRIB2 3'UTR	F: CCTCGAGTCTTTGTTTTATTAGAAACTAGTGAAACAA<br>R: TTGCGGCCGCACATACTAGAGTCCACGTGTCACCT	560
TRIB2 3'UTR Mut	Fm: TACTTCAATATGCCCATGACTTGG<br>Rm: CCAAGTCATGGGCATATTGAAGTA	560
MTMR3 3'UTR	F：CCTCGAGTGTGTCTCAGGAAGGTGGTTCT<br>R: TTGCGGCCGCGCTACCATGTGGAACACTCAACTT	530
MTMR3 3'UTR Mut	Fm: GGTTATGAATGCCCATTCTTGGG<br>Rm: CCCAAGAATGGGCATTCATAACC	530

Table S2. miR-100-5p targets predicted by software.

Groups	Genes
Targets predicted by five software (miRDB, Targetscan 7.1, miRWalk, miRPathDB and StarBase)	EPDR1, SMARCA5, BAZ2A, MTMR3, AGO2, HS3ST3B1, TRIB2
Targets predicted by four software (StarBase, miRDB, miRPathDB and miRWalk)	RNF144B, ZNF197
Targets predicted by four software (StarBase, TargetScan, miRDB, miRWalk)	PPP3CA
Targets predicted by four software (StarBase, TargetScan, miRPathDB and miRWalk)	GRHL1, KDM6B, RASGRP3, SMARCD1, CLDN11, ZBTB7A, ICMT, ST5, SATB1
Targets predicted by four software (TargetScan, miRDB, miRPathDB and miRWalk)	HES7, ST6GALNAC4, AP1AR, CTDSPL, TAOK1
Targets predicted by four software (StarBase, TargetScan, miRDB and miRPathDB)	KBTBD8, ZNRF2, MBNL1, FGFR3, FZD8, MTOR, TRIB1, RAVER2

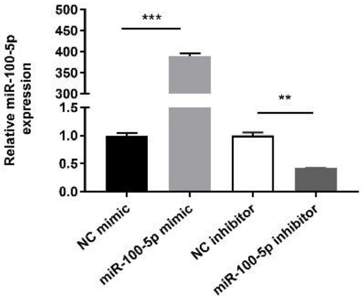

Figure S1 miR-100-5p detection in transient transfected THP-1 cells. THP-1 cells were transfected with oligonucleotides (NC mimic, miR-100-5p mimic, NC inhibitor and miR-100-5p inhibitor). Overexpression and inhibition efficiency of miR-100-5p were detected by qRT-PCR at 24 h post-transfection. Data are the mean ± SEM. **$p < 0.01$; ***$p < 0.001$.

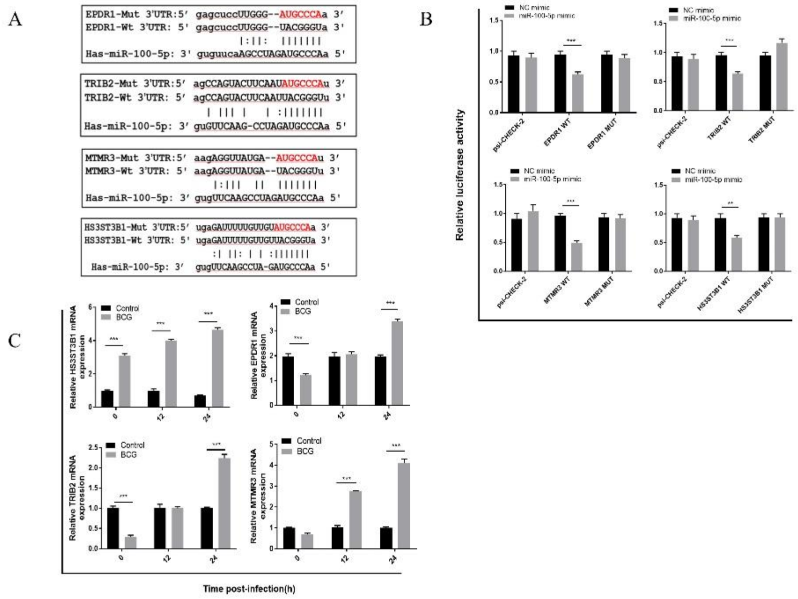

Figure S2 Screening and validation of miR-100-5p target genes. A The binding site of miR-100-5p with four target genes Wt or Mut 3′UTR as predicted by online prediction software. **B** Dule Luciferase Reporter assay was performed in the HEK 293T cells. The miR-100-5p mimic or NC mimic were co-transfected with Dule Luciferase Reporter plasmids and the ratio represents the Renilla to Firefly (Rluc/Fluc) ratio in miR-100-5p mimic vs. NC mimic. **C** PMA-differentiated THP-1 cells were infected with BCG at an MOI of 10 for 8 h using the uninfected cells as the control. Then, total RNA was extracted at 0, 12 and 24 hpi. The expression level of HS3ST3B1, TRIB2, MTMR3 and EPDR1was measured by qRT-PCR normalized with β-actin as the internal reference. The data are expressed as mean ± SEM. Statistical analysis was conducted with ANOVA, the significant differences is presented as ***$p < 0.001$.
